# An Overview of the Pathogenesis, Transmission, Diagnosis, and Management of Endemic Human Coronaviruses: A Reflection on the Past and Present Episodes and Possible Future Outbreaks

**DOI:** 10.3390/pathogens10091108

**Published:** 2021-08-30

**Authors:** Adeoye J. Kayode, Folasade O. Banji-Onisile, Ademola O. Olaniran, Anthony I. Okoh

**Affiliations:** 1 Applied and Environmental Microbiology Research Group (AEMREG), Department of Biochemistry and Microbiology, University of Fort Hare, Private Bag X1314, Alice 5700, South Africa; aokoh@ufh.ac.za or; 2 Wastewater Coronavirus Surveillance Laboratory, SAMRC Microbial Water Quality Monitoring Center, University of Fort Hare, Private Bag X1314, Alice 5700, South Africa; 3 Department of Microbiology, School of Life Sciences, College of Agriculture, Engineering and Science, University of KwaZulu-Natal, Durban 4000, South Africa; 220112317@stu.ukzn.ac.za (F.O.B.-O.); olanirana@ukzn.ac.za (A.O.O.); 4 Department of Environmental Health Sciences, College Health Sciences, University of Sharjah, Sharjah 555588, United Arab Emirates

**Keywords:** pandemic, SARS-CoV, MERS-CoV, SARS-CoV-2, aerosols, vulnerable individuals, globalization, coronaviruses, vaccine

## Abstract

The outbreak of the 2019 coronavirus pandemic caught the world by surprise in late 2019 and has held it hostage for months with an increasing number of infections and deaths. Although coronavirus was first discovered in the 1960s and was known to cause respiratory infection in humans, no information was available about the epidemic pattern of the virus until the past two decades. This review addresses the pathogenesis, transmission dynamics, diagnosis, management strategies, the pattern of the past and present events, and the possibility of future outbreaks of the endemic human coronaviruses. Several studies have described bats as presumptive natural reservoirs of coronaviruses. In essence, the identification of a diverse group of similar SARS coronaviruses in bats suggests the possibility of a future epidemic due to severe acute respiratory syndrome (SARS-like) coronaviruses originating from different reservoir hosts. The study also identified a lack of vaccines to prevent human coronavirus infections in humans in the past, however, the recent breakthrough in vaccine discovery and approval for emergency use for the treatment of Severe Acute Respiratory Syndrome Coronavirus 2 is commendable. The high rates of genomic substitution and recombination due to errors in RNA replication and the potential for independent species crossing suggest the chances of an entirely new strain evolving. Therefore, rapid research efforts should be deployed for vaccination to combat the COVID-19 pandemic and prevent a possible future outbreak. More sensitization and enlightenment on the need to adopt good personal hygiene practices, social distancing, and scientific evaluation of existing medications with promising antiviral effects against SARS-CoV-2 is required. In addition, intensive investigations to unravel and validate the possible reservoirs, the intermediate host, as well as insight into the ability of the virus to break the species barrier are needed to prevent future viral spillover and possible outbreaks.

## 1. Introduction

The increase in the emergence and reemergence of contagious pathogens is a serious threat to public health globally [1]. Historically, infectious pathogens are among the leading causes of morbidity and they account for approximately 22% of all deaths annually [2]. Among these pathogens, the viral counterparts are increasingly endangering humans, probably because of the increased human-animal interface, easy transfer of viruses from their wild reservoirs to farm animals, rapid encroachment of natural habitats for wild fauna to meet the demand for food by the rapidly growing human population as well as the rapid encroachment of natural habitats due to urbanization [3].

**Figure 1:**
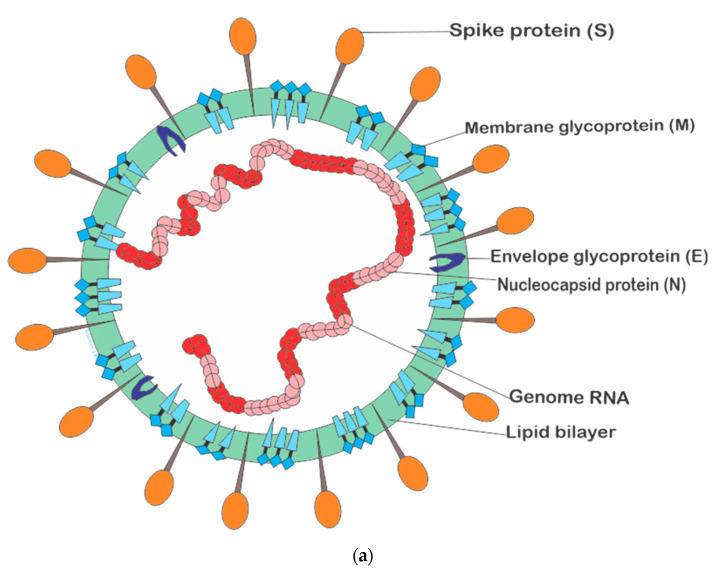

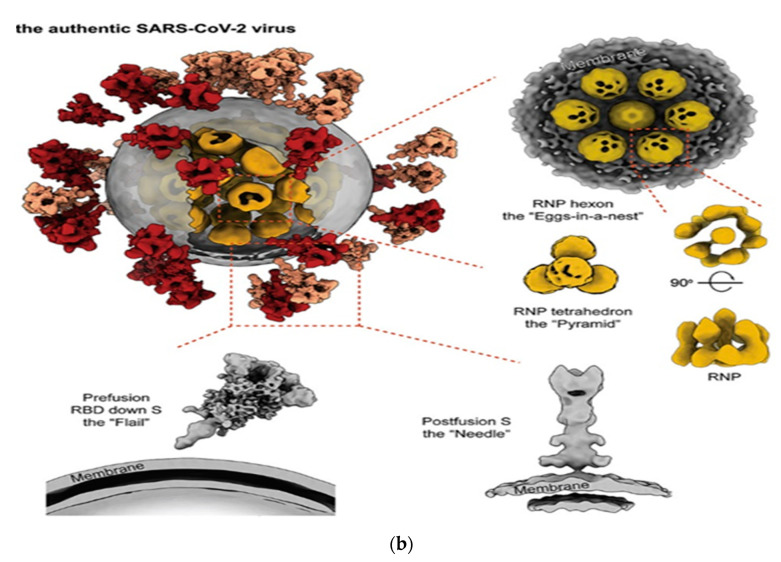
(**a**). The general structural representation of human coronaviruses. (**b**) Molecular structural description of SARS-CoV-2 [7] Key: RNP (ribonucleocapside), RBD (receptor binding domain).

Coronaviruses are a diverse viral group that infects humans and a wide variety of wildlife, including cattle, camels, pigs, bats, and birds. Coronaviruses (CoVs) can cause infections in various systems, such as the respiratory, hepatic, and gastrointestinal tracts. accompanied by mild or severe neurological illnesses that could eventually lead to death [4]. Coronaviruses are non-segmented, positive-sense single-stranded RNA enveloped viruses with genome dimensions of about 26–32 kilobases, usually the largest RNA viral genome known [5]. This viral group possesses a nucleocapsid comprised of genome RNA and phosphorylated nucleocapsid (N) protein embedded in the phospholipid bilayer protected by two distinct protein spikes: the hemagglutinin–esterase (HE) found in some coronaviruses and spike glycoprotein trimmer (S) found in every coronavirus. The envelope glycoprotein (E) and the membrane glycoprotein (M) and protein (type III glycoprotein) are situated amid the spike protein in the viral envelope [6], as represented in Figure 1a.

Recent studies unveiled the new structural architecture of the novel SARS-CoV-2 (Figure 1b) with a detailed description of the native structural appearance of the S proteins both in the perfusion and the postfusion conformations. The structures were obtained with the aid of cryo-electron tomography (cryo-ET) and subtomogram averaging (STA) detailing the overall architecture of SARS-CoV-2 and the configuration of the ~30 kb long single-stranded RNA (~80 nm diameter) in the lumen [7,8].

Human coronaviruses (HCoVs) represent the major group of coronaviruses linked to diverse respiratory illnesses of various severity including bronchitis, common cold, and pneumonia. Currently, human coronaviruses are among the most rapidly evolving viruses due to high rates of recombination and substitution of genomic nucleotides [9,10]. There have been three major outbreak episodes over the last two decades connected to coronaviruses originating from animals causing severe infections in humans: China and Hong Kong in 2002–2003, Saudi Arabia in 2012 [11], and the current ongoing pandemic. As such, this study reviewed published scholarly articles on human coronaviruses and appraised the state of existing knowledge on the pathogenesis, transmission, diagnosis, management, and control of human coronaviruses. It also reflects on past and current episodes of the infection and where research efforts could be deployed for possible discoveries that could serve as feasible countermeasures to the current ravaging pandemic and possible future outbreaks.

## 2. Classification of Human Coronavirus (HCoVs)

The genus coronavirus belongs to the order *Nidovirales*, family *coronaviridae*, subfamily *coronavirinae* [5]. The virus was named several decades ago, having coined “*coronavirus*” from the Latin word “*corona*” meaning “crown or halo” which describes the structural appearance of the virus under an electron microscope. The viral spikes (S) and the peplomers occupying the surface determine the appearance and host tropism [5,12]. Coronaviruses were categorized into four genera, including *Alpha, Beta, Gamma,* and *Delta coronaviruses*. The genus *Betacoronavirus* was assigned to four (A, B, C, and D) distinctive lineages based on serological as well as genomic proofs [13,14]. Currently, about 30 coronaviruses that infect humans and animals exist, and six of these viruses have been recognized to infect and inflict respiratory diseases in humans [6]. Among the HCoVs, HCoV-229E, and HCoV-NL63 belong to the *Alphacoronavirus,* while members of the *Betacoronavirus,* HCoV-OC43 and HCoV-HKU1, were assigned to lineage A, SARS-CoV belonged to lineage B, and MERS-CoV belonged to lineage C [5]. On 12 December 2019, the first 27 new viral pneumonia cases caused by a novel coronavirus (seventh member of the HCoVs belonging to the genus *Betacoronavirus*) was announced by the Wuhan Municipal Health Commission in Wuhan, China [15]. The virus was named the 2019 new coronavirus (2019-nCoV) or HCoV-19 by the World Health Organization. After much investigation, the virus was officially described by the International Committee on Taxonomy of Viruses (ICTV) as “Severe acute respiratory syndrome coronavirus-2 (SARS-CoV-2)” [16,17] based on information unveiled by scientific clinical research.

## 3. Pathogenesis of Human Coronaviruses

Coronaviruses are common in bats and are widely distributed in many other animal groups, including mice, cats, dogs, pigs, horses, birds, whales, and humans [18]. They may cause enteric, respiratory, hepatic, or neurologic disorders with varying severity in humans and animals [18]. The unusual emergence and reemergence of these viruses were attributed to the high recurrence of RNA recombination, the large RNA genome, and the infidelity of the RNA-dependent RNA polymerase [19]. Coronaviruses are considered zoonotic and highly pathogenic viruses and have been implicated in three major regional and global outbreaks in the last two decades, including Severe Acute Respiratory Syndrome Coronavirus, Middle East Respiratory Syndrome Coronavirus together with Severe Acute Respiratory Syndrome Coronavirus-2 accountable for the current pandemic [16].

### 3.1. Severe Acute Respiratory Syndrome Coronavirus (SARS-CoV)

The first human coronavirus outbreak was heralded in Southern China in November 2002. It spread rapidly across 33 countries on five continents, leading to about 8422 confirmed cases and over 916 deaths after the outbreak in June 2003, with 11% fatalities [11,20,21]. SARS-CoV contributed about 22% and 22.8% of all confirmed cases in Hong Kong and Guangdong, respectively, during the 2002–2003 outbreak [22,23]. This etiological agent (SARS-CoV) was discovered in the Chinese horseshoe bat (*Rhinilophidae*) as a natural reservoir [24]. The first cases of the SARS-CoV outbreak in Guangdong were connected to restaurant workers trading wild animals as exotic foods. History revealed that the workers had no contact with any other animals except rats, suggesting that rats could have played an important role in the transmission of the virus from bats to humans [25,26]. Serological studies confirmed antibody production against SARS-CoV and other related viruses and were present at a higher ratio among market traders compared to other populations during the 2002–2003 outbreak [14]

Further investigations revealed that raccoon dog and civet samples from the wildlife food market in Shenzhen, Guangdong province, tested positive for viral RNA [14], suggesting *Himalayan* palm civets as the probable secondary hosts [14,27,28,29]. People infected with SARS-CoV develop flu-like symptoms, dry cough, fever, headache, hypoxemia, dyspnea, and pneumonia after an incubation period of four to six days. Also, acute respiratory distress syndrome has been reported in severe cases [25]. SARS-CoV can infect many organs and cause systemic disorders. Symptoms become worse as the virus is cleared, suggesting that an aberrant immune response could underlie SARS-CoV pathogenesis [20]. Another sporadic outbreak was reported in the same region in late 2003–2004 by different isolates, which indicates the chances for independent species-crossing events, the possibility of a future outbreak and that SARS-like coronaviruses originating from different reservoir hosts may cause future epidemic episodes at different times and regions based on the location and distribution of the transmitting host [26]. The detection of a diverse group of SARS-like coronaviruses in bats justifies the chance of recurring future outbreaks [26].

### 3.2. Middle East Respiratory Syndrome Coronavirus (MERS-CoV)

About 10 years after the SARS-CoV outbreak, another outbreak of MERS-CoV was described in June 2012 and on 3 November 2013, which emerged in Saudi Arabia [30]. The first case of the outbreak was linked to a 43-year-old man admitted to Abdulaziz University Hospital Jeddah who previously had contact with a herd of nine camels. MERS-CoV causes infections resulting in respiratory tract illnesses accompanied by rhinorrhea, cough, severe shortness of breath, malaise, and fever in patients [31]. Two significant outbreaks were caused by MERS-CoV in Saudi Arabia (2102 cases, 780 deaths, 37.10% fatality) in 2012 [32] and South Korea in 2015, and approximately 80% of the cases originated from Saudi Arabia [33]. Up until November 2019, globally confirmed cases rose to 2494, 858 deaths, and 34.40% fatalities across 27 countries [27,31,34]. Severe cases were more pronounced in the elderly, particularly those with comorbidities [35]. Epidemiological studies suggest that human infections depend on multiple zoonotic transmissions from an animal reservoir [31]. Akin to SARS-CoV, genomic sequences indicated that bats were the primary reservoir of MERS-CoV [31], and dromedary camels were later identified as the intermediate hosts [35,36]. This was supported by serological studies revealing the presence of MERS-CoV cross-reactive antibodies in Egyptian, Canary Island, and Oman camels. However, further studies revealed that MERS-CoV isolates sampled from camels and humans had identical genome sequences; this serves as evidence of transmission of SARS-CoV from camel to humans by close contact [31]. Secondary transmission (human to human) was established both in household and health care environments [31,32,37].

### 3.3. Severe Acute Respiratory Syndrome Coronavirus 2 (SARS-CoV-2)

An interestingly new coronavirus, like other coronaviruses of the genus *Betacoronavirus*, linked to a common potential reservoir, intermediate and final hosts emerged in China in 2019. It is ranked next to the Spanish influenza pandemic, which claimed about 40 million lives globally in 1918–1919 [38]. SARS-CoV-2, compared to SARS-CoV as well as MERS-CoV, has higher transmissibility and infectivity but a low fatality average of 2–4% (although this could vary between countries and across ages) [39,40]. This constitutes a major challenge to the treatment and prevention of SARS-CoV-2. According to data retrieved from Worldometer [41] on 11 July 2021 (13:24 GMT), about 187,410,774 cases, 171,361,359 recoveries and 4,045,522 deaths caused by the SARS-CoV-2 pandemic have been recorded and are still counting (Table 1).

Patients infected with SARS-CoV-2 show pneumonia symptoms accompanied by high fever, malaise, dry cough, sore throat, fatigue, sneezing, body pain, and difficult breathing [11,42]. Several studies have investigated the origin of the virus after recording the first cases, identifying that most of the patients were previously exposed to wildlife in recent history at the popular Wuhan Seafood Market, China, known for wholesales of snakes, bats, poultry, and other wildlife [15]. The source of the novel virus was suspected to be bats [17]. Studies on SARS-CoV-2 genomic sequences revealed 79.5% and 96% similarity to SARS-CoV genome and bat coronavirus, respectively (SARSr-CoV-RaTG13) [15,17]. However, there are reports that no epidemiological link exists between the first patient hospitalized on 16 December 2019 and 2 January 2020 and cases detected later, meaning that the first (13 out of the 41) cases had no contact with the Wuhan Market [15,16]. Daniel Lucy further emphasized that SARS-CoV-2 may have about 14 days’ incubation period such that the first described case was detected on 1 December 2019. There is a possibility of the first contagion of humans taking place in November or earlier and spreading unnoticed among people in Wuhan until cases from the seafood market were detected in late December 2019 [15,43]. Another study carried out a phylogenetic study on the whole viral genome and identified that SARS-CoV-2 was a more closely related coronavirus (SARS) class isolated from Chinese bats [44]. A comprehensive analysis of RNA genome sequences in combination with the relative usage of synonymous codon (RSCU) carried out amongst various animal species found SARS-CoV-2 as a recombinant virus between bat CoVs and other CoVs. However, the snake was pointed to as the suspected reservoir of the virus due to high RSCU bias similarity to snake [45]. Genome sequences of the viral samples obtained from patients across countries were identified with mutations and deletions, indicating genetic diversity and evolution [46]. Another study led by Zhu deployed a deep learning algorithm to analyze the gene sequences of SARS-CoV-2 and other coronaviruses to predict the possible host of the virus. This investigation revealed that minks and bats could be the potential reservoirs, and minks could be the intermediate hosts of the novel coronavirus [47].

Further investigations revealed high similarity (99%) between SARS-CoV-2 isolates obtained from pangolin and the current novel SARS-CoV-2 isolates through molecular biology detection, macro-genomic sequencing, and electron microscopy, which identified pangolin as a potential intermediate host [23]. Generally, SARS-CoV-2 exhibits a similar pattern of infection to other human CoVs, especially SARS-CoV, Bat SARS-like coronavirus, and MERS-CoV. Bat CoVs had the most similar infection pattern to SARS-CoV-2, SARS-CoV and MERS-CoV after comparing with CoVs in other vertebrates, but CoVs from mink hosts shared the most similar infection pattern with the (CoVs) novel coronaviruses [15]. However, more studies are necessary to investigate the origin of the SARS-CoV-2 virus to establish the natural and intermediate host.

#### Mutant Variants of SARS-CoV-2

Coronaviruses contain nearly 30,000 RNA sequences carrying information that enable them to infect cells and replicate by hijacking the cellular mechanisms of host cells to make new viral copies. During replication in infected cells, errors (mutations) occasionally occur giving rise to new coronavirus variants. Globally, there are several SARS-CoV-2 variants categorized by the CDC as variants of concern (Table 2), variants of high consequence, and variants of interest. The major concerns about the emergence of the new variants to human health are higher transmissibility, increased ability to evade vaccine-induced immunity, ability to evade natural immunity, and disease severity.

The B.1.1.7 variant was first detected in the United Kingdom on 14 December 2020 and found with 23 mutations and 17 amino acid changes. The P681H mutation helps to efficiently create new spike proteins while the H69-V70 and Y144/145 deletions alter the spike shape and facilitate antibody evasion. The mutant variant B.1.351 (23 mutations and 17 amino acid changes) was detected in South Africa and the Brazilian mutant variant was detected in Brazil (approximately 35 mutations with 17 amino acid changes). All three variants have the N501Y mutation and have been reported in many countries globally. Mutation in these variants occurs as a result of changes in the amino acid asparagine-to-tyrosine at point 501 in the receptor-binding domain of the spike protein. Furthermore, additional receptor binding domain mutations E484K and K417T were found in variants P.1 and 501Y.V2 known to increase the affinity of the RBD to the angiotensin-converting enzyme 2 (ACE2) receptor [48].

The G/452R.V3 variant was identified in Maharashtra, India on 5 October 2020. As of May 2021, three sublineages including B.1.617.3 (first detected in October 2020) relatively uncommon compared with the B.1.617.1 and B.1.617.2 both detected in December 2020. The B.1617.2 was classified as a variant of concern responsible for the current devastating Indian second wave. Mutation in this variant occurs in the spike protein genes of SARS-CoV-2 causing substitutions in the amino acid sequences. The B.617 genome has between 13 and 17 substitutions. The D614G is an aspartic acid-to-glycine substitution which occurs at position 614 similar to other highly transmissible strains. E484Q, a glutamate-to-glutamine substitution occurs at position 484 and confers a stronger binding potential to the human ACE2 receptor and the ability to evade host immune response. The E484Q is not found in the B.1.612.2 genome. Also, the L452R, a leucine-to-arginine substitution confers a stronger affinity to the ACE2 receptor and decreased the ability of the immune system to recognize it. Furthermore, P681R, a proline-to-arginine substitution facilitates the cleavage of the S precursor protein to the active S1/S2 configuration and boost cell level infectivity of the variant. Variant B.1.617.3 and B.1.617.1 shares the E484Q and L452R mutation, B.1.617.2 has the T478k mutation not found in B.1.617.1 but lacks E484Q mutation [49]

### 3.4. Other Human Coronaviruses

In the past two decades, three of the seven HCoVs (SARS-CoV, MERS-CoV as well as SARS-CoV-2) have been implicated in major outbreaks, which were predicted to have spilt from the Chinese horseshoe (*Rhinolophiae*) bat. In 1967, HCoV-OC43 was isolated at the Laboratory of Viral Diseases, National Institutes of Health, Bethesda, Maryland [50] and HCoV-229E was isolated at the Laboratory of Infectious Diseases, National Institute of Allergy and Infectious Diseases, National Institutes of Health, Bethesda, Maryland [51]. Comparisons of the genetic elements of bovine coronavirus (BCoV) and HCoV-OC43 revealed that HCoV-OC43 might have emerged from a zoonotic transmission from bovine to human [9], and HCoV-229E was stated to share a most recent common ancestor (MRCA) with the bat (*Hipposieros caffer ruber*) in Ghana by molecular clock analysis [52]. The close link between bats and human CoVs led to the conjecture that all humans and perhaps mammalian coronaviruses might emerge from bats [53,54]. These viruses (HCoV-229E and HCoV-OC43) have been reported to be accountable for about 10–30% of the most common colds in winter and early spring [9]. HCoV-NL63 was isolated and identified in 2004 from a 7-month old baby diagnosed with conjunctivitis and bronchitis during winter in the Netherlands [55]. Another sample from an 8-month-old baby suffering from pneumonia, including six other individuals, tested positive for the same virus [49]. Furthermore, strains of HCoV-NL63 with different genetic markers were isolated in France from hospitalized children diagnosed with respiratory tract infection from November 2002 to April 2003 [56]. From this time, a report showed that HCoV-NL63 was usually detected in respiratory samples (1–9.3%) collected from different countries, which indicates its worldwide distribution and suggesting that HCoV-NL63 is a novel etiological agent of respiratory tract illnesses [9,57]. The potential reservoir of this virus has not been described, although phylogenetic proof indicates that the virus emerged from HCoV-229E 1000 years ago and has infected humans for centuries [53,58]. HCoV-HKU1, another HCoVs, was reported as part of the virus responsible for acute respiratory tract infection (ARTI). It was first reported in January 2005 after detection in a pneumonia patient. Few cases of infections caused by HCoV-HKU1 were reported, including two cases in Hong Kong in 2005 and one other case [59,60]. Another study carried out on a population of 324 specimens obtained during winter indicated eight positive cases for HCoV-HKU1, of which all were children of less than four years excluding a 40-year-old adult positive for ARTI in Sweden [61]. All HCoVs, belong to the genus *Alphacoronavirus* and *Betacoronavirus* [5]. Several studies investigated the origin, natural reservoir, and intermediate hosts of coronaviruses. Woo and his colleagues discovered that the diversity of coronaviruses found in bats are like those commonly found in birds. The vast majority of bat CoVs belong to the *Alpha* and *Beta* coronaviruses, while bird CoVs belong to the Gamma and Delta coronaviruses. The outcome of the study identifies bats as the genetic source of Alpha and Beta CoVs and birds as the genetic source of *Gamma* and *Delta* CoVs, agreeing with the evolutionary model employed for the study [14].

Many emerging viruses associated with human diseases were traced to bat as the major reservoir hosts. However, a few species (~36 out of 1100) have been investigated [62,63,64] and they harbour RNA viruses that share similarities in their sequences [53]. Considering the abundance of CoVs in several bat species worldwide may likely be an indication that bats are especially suited by nature to keep CoVs [24,52,53]. Increasing evidence suggests that mammalian CoVs may have emerged from bats [54,65] especially human CoVs [53]. Another study attempted to investigate the ability of CoVs to infect multiple mammalian hosts by cultivating human CoVs on immortalized U.S. bat species cell lines. The study established the zoonotic-reverse zoonotic cycles, which enable coronaviruses to keep up the viral community in many hosts, evolving interestingly new recombinant viruses having the viral genes originating from humans and animal coronaviruses and transported at a later time back into humans [53]. This also suggests that HCoV-NL63 might have emerged from bats and crossed species barrier to infect humans [53].

## 4. Pathogenic Mechanisms and Susceptible Groups

### 4.1. Pathogenic Mechanisms of Human Coronaviruses (HCoVs)

The infection cycle of human coronaviruses is usually preceded by viral attachment to a specified receptor and human cellular entry aided by surface glycoprotein spikes. Naturally, all CoVs possess specific genes downstream of the Open Reading Frame (ORF) region, which carries the genetic information required for the viral replicative process, including nucleocapsid and spike making [66]. The viral spikes (S), occupying the superficial layer comprises two active subunits, bulb (S1) for binding the receptors and the stalk (S2) for membrane fusion. Clearly defined interaction that occurs between the connected receptor and S1 activates a radical structural alteration in the S2 subunit, facilitating the bonding between the cellular membrane and the viral envelope and the discharge of the nucleocapsid into the cytoplasm [5,67]. The binding region of the receptor is not firmly attached among viruses; this enables the virus to infect more than one host [20]. SARS-CoV and MERS-CoV recognize exopeptidase, while other CoVs, in most cases, recognize carbohydrates or aminopeptidases [68]. The CoVs system of entry is dependent on cellular protease including cathepsins, transmembrane protease serine 2 (TMPRSS2), and human airway trypsin-like protein (HAT) that breaks the spike protein and initiates further penetration [69,70]. SARS-CoVs and HCoV-NL63 require the ACE2 receptor, and in essence, infect bronchial ciliated cells and type II pneumocytes [47] while MERS-CoV employs dipeptidyl peptidase 4 (DPP4/CD26) and infects unciliated bronchial epithelial cells and type II pneumocytes [68,71,72]. SARS-CoV-2 owns a quintessential structure with spike protein and membrane proteins, such as the nucleoprotein, RNA polymerase, papain-like protease, helicase, glycoprotein, 3- chymotrypsin-like protease, ancestry proteins, and polyproteins [17]. The SARS-CoV-2 spike protein has a three-dimensional shape in the RBD region which keeps the van der Waals force [23] and has a higher affinity (about 10–20 times) to ACE2 in humans compared to SARS-CoV, which in part explains more chances of human-to-human transmission [47]. The critical 31 lysine residue in human ACE2 identifies the residual 394 glutamine in the RBD domain of SARS-CoV-2 on the human ACE2 receptor [27].

### 4.2. Vulnerable Individuals to Coronaviruses Infection

Based on demographic data, human CoVs infect virtually all age categories with a 1:1.25 male-to-female ratio and the possibility of reinfections are common [9,23,73,74]. The infection can be mild and subclinical and may affect the lower respiratory tracts [75,76]. Severe cases are mostly recorded in elderly persons, particularly those with comorbidities [35]. SARS-CoV was estimated to have an incubation period of 1–4 days [77] and a higher duration of incubation (10 days) may be observed in a few patients [78]. SARS-CoV infection has a latency period of 4 days, an average interval of 3.8 days from the initial of symptom manifestations to hospital admission, and 17.4 days’ in-between hospital admission and death [77]. For MERS-CoV, the infection median latency was 7 days [79]. Based on epidemiological investigation, the elderly within age 75 (median age at death) are the most susceptible group to SARS-CoV-2 infection, most of the deaths are linked to patients with histories of surgical procedures or comorbidities before admission [80]. The median period of incubation of SARS-CoV-2 ranged from 0–24 days (median of 3 days) based on clinical features [80,81], and the latency period of the disease is estimated at 3–7 days and up to 14 days in some cases [1]. Throughout this incubation period, patients are contagious, and each case could infect an average of 3.77 people with uncertainties between 2.23–4 [74,82]. SARS-CoV-2 possesses a shorter median incubation period compared to SARS and MERS coronaviruses. Nevertheless, SARS-CoV-2 has a maximum latency period of 24 days based on a recent report, which suggests an increased risk of viral transmission [83]. However, people aged ≥ 70 have shorter (11.5 days) median intervals from the onset of symptoms manifestation to death compared to 20 days for patients under 70 years, suggesting that the progression of the disease is more rapid in the elderly compared with younger people [80]. With these observations, more attention and care should be given to the elderly because of their higher vulnerability to SARS-CoV-2.

## 5. Transmission of Human Coronaviruses

Previous epidemiological studies identified transmission routes, globalization, and urbanization as important factors for the widespread nature of viral infections [2,3] inclusive of CoVs [83].

### 5.1. Transmission Dynamics of Human Coronaviruses

Several studies have established the spread of coronavirus among humans [16,84]. Person-to-person dissemination is usually through aerosols or droplets of bodily fluids, such as mucus or aerosols from infected persons during sneezing or coughing [27,85]. Due to the high transmissibility of the virus, health workers are usually at higher risk due to frequent interactions with infected persons [74]. Cases of viral transmission from both symptomatic and asymptomatic patients to health workers were reported [86], but no cases of placental transmission in the foetus have been documented. Chen and his colleagues also confirmed the transmission of SARS-CoV from patients to health workers in Taiwan [87], especially from patients with high viral loads [88]. Transmission of MERS-CoV was also reported in about 27 countries globally, and so were numerous clusters of cases in Qatar, Saudi Arabia, United Arab Emirates, Tunisia, Jordan, France, and United Kingdom, among health workers, family members, and travellers [32,37,71,89,90]. Several studies established the spread of SARS-CoV-2 among humans [84]; this was corroborated by an investigation where five out of six members of a family, with travel history from Shenzhen to Wuhan but who did not visit the Wuhan Market on 29 December 2019 nor had any contact with animals, acquired SARS-CoV-2 infection. However, two of the patients had visited a Wuhan hospital, and one family member with no travel history to Wuhan who had contact with them was also infected [42]. Cases of person-to-person transmission of SARS-CoV-2 in the USA were also reported among people in close contact [91]. Furthermore, studies revealed that about 50–80% of the viral infection is transmitted by asymptomatic carriers [92] via natural aerosols; besides, saliva, urine, and stool samples tested positive for SARS-CoV-2 [12,83,84,93]. Transmission from person-to-person via mechanical aerosols as well as the nosocomial transmission was also reported during the 2002 through 2003 SARS-CoV outbreak [94]. Further investigations revealed that SARS-CoV was detected in stools and mechanical aerosols generated during flushing could also transmit the virus [94]. Recent research also revealed that SARS-CoV-2 was detected in semen, which suggests its possible transmission sexually [95]. Table 3 presents the comparisons of clinical manifestation, transmission routes, and the detection of HCoVs.

### 5.2. Factors Influencing the Transmission of Human Coronaviruses

Several factors contribute to the rapid spread of infectious diseases. The rapid spread of human coronaviruses outbreaks, such as the SARS-CoV, MERS-CoV, and especially the current SARS-CoV-2 pandemic, and the rapid spread across borders could be influenced by globalization, population growth, and urbanization [2,3].

#### 5.2.1. Globalization

Globalization exacerbates the risks constituted by the rapid spread of infectious diseases as it could lead to unexpected transmission of infectious agents with epidemic capacity within and across borders. The rapid increase in commerce and the ease of worldwide air travel amount to complications in controlling outbreaks at the earlier stage [3]. Several countries all over the world reported a high rate of imported cases among travellers from infected regions [98]. Person–to-person contact among travellers returning from China resulted in high rates of imported cases of SARS-CoV-2 by travellers using various means of transportation (taxis, trains, ships, planes) across all countries [94,98]. Person-to-person transmission of SARS-CoV was also detected among a household within six days and in patients with travel history to Guangdong, China [87].

#### 5.2.2. Population Growth and Urbanization

Current demographic trends reflect the possibility of a rise in the transmission of human coronaviruses globally. The largest fraction of the world’s population resides in urban areas compared to rural areas [3]. Rapid population increase, urbanization, shortage of adequate housing in urban settlements, weak health facilities, and ageing population encourage the rapid spread and community transmission of contagious infections [3,99]. A SARS-CoV outbreak reported in Amoy housing estate, Hong Kong that about 67% of infected persons had diarrhoea, such that the spread of the virus was suspected to be through mechanical aerosols generated by flushing [100]. Increases in human–animal interactions have always posed risks of the emergence of pathogen spillovers [101]. The switch in nature of this interchange due to increased factory farming to meet food demand and continuous encroachment of natural habitats could promote additional zoonotic infections [3]. Changes in climatic conditions due to intruding into the habitat of various disease vectors also play a significant role in propelling the transmission of pathogens [102]. Considering the historical epidemiology of human coronaviruses, these factors might have contributed to the sporadic and global outbreak of these viral pathogens in the last two decades. The outbreak of SARS/MERS together with SARS-CoV-2 was first reported in Guangdong, China; Saudi Arabia (Middle East); and Wuhan, China, respectively, all hosting a human population of about ten to a hundred million residents in the respective cities.

## 6. Diagnosis and Treatment of Human Coronaviruses

### 6.1. Sample Collection

The rapidity and accuracy of laboratory testing procedures are largely dependent on appropriate sampling from patients, including the diagnosis of viral respiratory infections. Numerous studies revealed that human coronaviruses were detected in various samples from the upper and lower respiratory systems, such as the nasal nasopharyngeal (NP), throat, bronchial fluid, and sputum [103,104]. Samples of nasopharyngeal aspiration are also suitable for detecting HCoVs [22]. Recently, Wang and his colleagues reported that NP swabs significantly yielded more SARS-CoV-2 RNA (63%) compared to oropharyngeal (OP) swabs in China [13], which justifies the US Centre for Disease Control’s recommendation for the NP swabs for detecting SARS-CoV-2 RNA. However, it was advised that if OP samples of lower priority were collected, it should be done alongside NP swabs and transported in a universal viral transport medium [22]. Cheng et al. recommended collecting and testing bronchoalveolar fluid (BAL), sputum, and both lower and upper respiratory samples to achieve the most sensitive SARS-CoV, MERS-CoV detection [105], and SARS-CoV-2. The saliva test for SARS-CoV-2 was also developed [106]. Blood, urine, and stool samples could also be considered for detecting SARS-CoV and MERS-CoV, but they are less reliable compared to respiratory samples [105,107,108]. However, frequent detection of SARS-CoV RNA from faeces about two weeks after the onset of symptoms was reported [105]. Respiratory specimens obtained within the first few days of symptom onset were recommended for the most sensitive and reliable detection of the endemic HCoVs [105,109].

### 6.2. Clinical Diagnosis

Epidemiological history, clinical manifestations, and other supplementary examinations, which operate on detection of viral nucleic acid, immune recognition techniques (POCT—point-of-care testing of IgM/IgG) are often employed in the clinical diagnosis of HCoVs. Other assays including ELISA (enzyme-linked immunosorbent assay) or indirect immunofluorescence assay, whole blood samples, and computerized tomography (CT) scan are usually employed in the diagnosis of HCoVs [90,91]. Detection of HCoVs is often done by RT-qPCR (reverse transcriptase quantitative polymerase chain reaction) as well as deep sequencing approaches [22,73,110]. However, RT-qPCR is more routinely used for the detection of HCoVs in respiratory and blood samples due to high cost and limited access to sequencing facilities [111]. Although the detection of HCoVs by this method (nucleic acid detection) is known for its high sensitivity and specificity (gold standard) [112], there may be false-positive results [110]. Supplementary examinations are important for the diagnosis of SAR-CoV-2 in combination with symptoms and epidemiological history of patients [110]. MERS-CoVs are usually detected on clinical samples using a pan-CoV RT-PCR (reverse transcription PCR) [14,113]. Detection of SAR-CoV RNA in nasopharyngeal or blood samples was based on reverse transcriptase-polymerase chain reaction (RT-PCR). Detection of antibodies to HCoVs antigens in the serum or whole blood samples are usually carried out using the enzyme-linked immunoassay (EIA) or indirect immunofluorescence assay [18].

CT scans, especially high-resolution CT (HRCT), were proposed by clinicians to supplement RT-qPCR negative results for patients with high clinical suspicion because of their sensitivity [114]. CT scans have high clinical diagnostic use for the detection of MERS-CoV and SARS-CoV-2, but it may be difficult to distinguish other non-HCoV viral pneumonia due to abnormal CT images [110]. However, lung ultrasound (LUS) seems to be a valid non-ionizing alternative especially in the initial assessment of lung involvement in SARS-CoV-2 infection and severity of SARS-CoV-2 pneumonia [115]. LUS could favourably be a substitute when CT devices are not available. Despite the satisfying sensitivity of both CT and lung ultrasound diagnosis techniques, they have low specificity.

Application of rapid antigen testing, such as the current POCT of IgM/IgG and the ELISA technology, which targets viral antigens or antibodies, may provide fast and cost-effective detection of HCoVs mostly at the early stage of infection. Zhang and colleagues recently developed a new diagnostic procedure called STOP (SHERLOCK Testing in One Pot) for SARS-CoV-2 rapid detection within 1 h [116]. Although the technology is still awaiting approval for clinical use, it is expected to remedy the high demand for a rapid test due to the rapidly increasing global spread of SARS-CoV-2.

## 7. Treatment of Human Coronaviruses

In the recent months of the COVID-19 pandemic outbreak, some drugs were engaged for SARS-CoV-2 treatment, especially those identified by cell-based in vitro assay or molecular docking with the potential to inhibit SARS-CoV-2 main proteases, and were considered as potential therapeutic options. The drugs were identified targeting various stages of the SARS-CoV-2 life cycle. In this report, common drugs having clinical relevance against SAR-CoV-2, including remdesivir, favipiravir, ritonavir/lopinavir, ivermectin and chloroquine/hydroxychloroquine, were employed for the treatment of SARS-CoV-2 infections before vaccine approval for emergency treatment of SARS-CoV-2.

### 7.1. Therapeutic Agents Inhibiting Virus and Cell Membrane Fusion

#### Chloroquine/Hydroxychloroquine

In vitro assay also projects the efficacy of chloroquine to inhibit SARS-CoV-2 [117,118]. Chloroquine and hydroxychloroquine are known drugs that interfere with SARS-CoV-2 cellular entry by inhibiting glycosylation and elevating endosomal pH but had suffered controversies for the treatment of COVID-19 infection; however, they were previously used for the prevention and treatment of malaria [112,118,119]. Chloroquine could practically inhibit SARS-CoV-2 through its immunomodulatory activity and evidence of reducing viral replication [118,120,121]. Likewise, a combination of azithromycin and hydroxychloroquine was discovered to possess a significant synergetic effect to decrease viral load and early recuperation [122]. However, WHO in a report recommended against using hydroxychloroquine or chloroquine in addition to the usual treatment given to patients with COVID-19 irrespective of the disease severity or duration of symptoms. Based on available data analyzed, there was no evidence that hydroxychloroquine and chloroquine reduced mortality or mechanical ventilation and may not reduce the duration of hospitalization [123]. Moreover, there was potential evidence for the need for mechanical ventilation and the risk of death with hydroxychloroquine. The effect on other less important outcomes, including time to symptom resolution, admission to hospital, and duration of mechanical ventilation, remains uncertain. Furthermore, hydroxychloroquine may increase the risk of diarrhoea and nausea or vomiting, a finding consistent with evidence from its use in other conditions. Diarrhoea and vomiting may increase the risk of hypovolaemia, hypotension, and acute kidney injury, especially in settings where healthcare resources are limited. Whether and to what degree hydroxychloroquine increases the risk of cardiac and neurotoxicity, including life-threatening arrhythmias, when used in patients with COVID-19 is uncertain [123].

### 7.2. Viral Protease Inhibitors

#### 7.2.1. Lopinavir/Ritonavir

Lopinavir, ritonavir, and enfuvirtide are therapeutic drugs (protease inhibitors) often used in combination therapy for HIV treatment [124,125] but have been found to possess inhibitory effects against the coronaviral protease 3CL [118,126]. However, data available were not sufficient enough to support the use of lopinavir–ritonavir in treating COVID-19 due to insignificant evidence of its efficacy and adverse reactions [127], such as gastrointestinal disorders. A recent report from WHO did not recommend using lopinavir–ritonavir in addition to the usual care for the treatment of patients with COVID-19 regardless of disease severity [123]. The outcome of an international standing Guideline Development Group’s investigation on the claim of lopinavir–ritonavir efficacy against SARS-CoV-2 revealed a lack of evidence on improved patient-important outcomes such as reduced mortality, need for mechanical ventilation, time to clinical improvement, and others. Other protease inhibitors and antivirals tested against SARS-CoV-2 main protease and protein spike have been proven to be effective and could serve as a potential antiviral agent for COVID-19 infection [125]. Umifenovir, a non-protease inhibitor, inhibits membrane fusion by targeting the interaction between the S protein and ACE2 [119].

#### 7.2.2. Ivermectin

Ivermectin was considered as an enigmatic multifaceted “wonder” drug in 2017 and has been studied since 1946 against avid diphtheria [128]_._ Ivermectin dissociates the preformed IMP α/β1 heterodimer known for transporting the bulk of viral proteins [129] essential for the replication cycle of and inhibition of the host’s antiviral responses [118]_._ Reports show that ivermectin demonstrated in vitro antiviral activity against SARS-CoV-2 clinical isolate with a single dose was able to control viral replication within 24–48 h [118,130]. Considering the safety profile of ivermectin and the lack of compelling evidence of its efficacy from SARS-CoV-2 treatment, the WHO did not approve it for treatment in patients regardless of disease severity or duration of symptoms [123]. Some other non-structural proteins, such as papain-like protease (PLP) and chymotrypsin-like (3CL-like protease, 3CLpro), play an important role during the replication of coronaviruses and inhibiting the innate immune responses [110,131]. Other attractive choices of 3CLpro, such as flavonoids [132] and cinanserin [133], and PLP inhibitors, such as diarylheptanoids [134], are other potential choices that could be used as an agent against SARS-CoV-2. Blocking the S protein and ACE as a functional receptor that moderates the entry of SARS-CoV-2 into the cell is also an important approach against SARS-CoV-2 disease [135]. The indole derivative molecule (Arbidol) presented a better therapeutic effect in randomized control studies for SARS-CoV-2 treatment and could decrease the incidence of severe cases significantly [83]. The neuraminidase inhibitors and peptide EK1 could have potential possibilities for SARS-CoV-2 treatment [136].

### 7.3. RNA Dependent RNA Polymerase Inhibitors

#### 7.3.1. Remdesivir

Antiviral drugs, such as remdesivir (an adenosine analogue), have been shown to be promising antiviral agents against various RNA viruses by targeting the RNA-dependent RNA polymerase as well as blocking viral RNA synthesis [118] in SARS-CoV and MERS-CoV infections in primate models [137], mice [138], and cultured cells [139]. It was experimented for treating Ebola virus infection [119]. In vitro assay also revealed the efficacy of remdesivir to inhibit SARS-CoV-2 [118]. The first intravenous administration of remdesivir by the Washington Department of Health revealed its prospects as an antiviral agent against SARS-CoV-2 [140]. Studies have also revealed that severe adverse effects, such as a reversible increase in transaminases with possible kidney damage, may arise as a possible health consequence of remdesivir [118,138]. A recent report by the WHO also suggests against administering remdesivir in addition to usual care giving to patients hospitalized with COVID-19 regardless of disease severity not necessarily because remdesivir has no benefit. This was based on a lack of possible evidence-based on currently available data on its effect on mortality, the need for mechanical ventilation, time to clinical improvement, and other patient-important outcomes [123].

#### 7.3.2. Favipiravir

Favipiravir, similar to remdisivir, works as an RNA-dependent RNA polymerase inhibitor with a structural resemblance to endogenous guanine [118,141]. Developed in 2002 to have selective inhibitory activity against influenza viruses and RNA viruses. High tolerance to favipiravir was reported but limited information is available on the safety level at a higher dose [118,142,143,144]_._ Adverse health effects include hyperuricaemia, a decrease in the number of neutrophils, an increase in transaminases, and diarrhoea [145]. Consequently, other nucleoside analogues (galidesivir, and ribavirin) may have the potential for clinical application against SARS-CoV-2 [146,147].

### 7.4. Antibody and Convalescent Plasma Therapy

Recently, Duan et al. reported significant improvements in laboratory parameters and clinical symptoms within three days of convalescent plasma transfusion in SARS-CoV-2 patients [148]. This motivated the idea of donating patients’ convalescent plasma for treating SARS-CoV-2, similar to trials on MERS-CoV [149]. Furthermore, the age of recombinant mAb (monoclonal antibody) is reasonably a direct path to inactivate SARS-CoV. Virus neutralizing antibodies (nAbs) induced by viruses or vaccines are instrumental in the control of viral infection. Several nAbs specific against SAR-CoV, MERS-CoV, and SARS-CoV-2 have been developed, including the single-domain antibodies (nanobodies, Nbs) or single-chain variable region fragments (scFv), monoclonal antibodies (mAbs), and functional antigen-binding fragments (Fab) [150,151]. Usually, these antibodies target the S protein, S1-RBD, S1-NTD, or the S2 region by preventing binding of RBDs to their specific receptors and interfering with the fusion of S2-mediated membrane or cellular entry, eventually hindering viral infection [152,153]. SARS-CoV specific human mAb (CR3022), which could be considered a prospective therapeutic candidate for SARS-CoV-2 infections after development, showed the capacity to bind strongly with the receptor-binding region (RBD) of SARS-CoV-2 [154].

Moreover, S309 antibody identified from memory B cells of an individual previously infected with SARS-CoV in 2003 was found potent in neutralizing SARS-CoV-2, SARS-CoV, and pseudoviruses by engaging the RBD of the S glycoprotein. The combination of S309 antibodies further enhanced the neutralization of SARS-CoV-2 and may limit the emergence of neutralization-escape mutants. The outcome of this investigation projects the use of this antibody for prophylaxis in individuals at a high risk of exposure or as post-exposure therapy to limit or treat severe diseases [155,156]. Other recently described potent neutralizing antibodies (nAbs) of two epitopes in the receptor-binding domain (RBD) and distinct non-RBD epitopes on the spike (S) protein were described by Rogers et al. Neutralizing antibodies (nAb) protected against disease in high-dose SARS-CoV-2 in Syrian hamsters. The study suggests a role for nAbs in prophylaxis and as a potential therapy for the COVID-19 pandemic [156].

Furthermore, Bin et al. reported the successful isolation and characterization of receptor-binding domain-specific monoclonal antibodies (206 RBD), a derivative of single B cells from eight individuals infected with SARS-CoV-2. The 206 RBD showed the capacity to potently neutralize SARS-CoV-2, by inhibiting viral engagement with ACE2 and eventually blocking viral entry. The antibody may be representative of the development of clinical interventions against SARS-CoV-2 [157]. Other mAb, such as CR3014, m396, and neutralizing SARS-CoV, could be a possible substitute for treating SARS-CoV-2 [158].

SARS-CoV nAbs currently available are those targeting the S protein, RBD, S2 subunit or the S1/S2 proteolytic cleavage site. Human neutralizing mAbs m396 and S230.15 isolated from individuals infected with SARS-CoV neutralized palm civet and human SARS-CoV infection through interaction with RBD and blocks binding between the viral RBD and cellular ACE2 receptor [151]. Other SARS-CoV human neutralizing antibodies (S109.8 and S227.14) were reported to have neutralizing activity against several humans, raccoon dog, and palm civet infectious clones [159], and a variety of human nAb 80R (scFv or mAb) neutralized SARS-CoV by blocking the RBD-ACE2 interaction [160]. SARS-CoV RBD specific mouse neutralizing mAbs were found to be potent in blocking RBD-ACE binding and in neutralizing infection in ACE2-transfected HEK293T cell [161]. Notwithstanding, none of the SARS-CoV nAbs have been evaluated in clinical studies.

MERS-CoV specific nAbs target the S protein [150,162], and a few specifically target epitopes in the S1-NTD and regions of the subunit [163]. Human Fabs or mAbs (MERS-27, MERS-GD27/MCA, m336), human mAbs (4C2 h, hMS-1), Nbs (HCAb-83 or NbMS10-Fc isolated from camel), and mouse mAbs (Mersmab1, D12 or 4C2, isolated from mice) were able to recognize RBD epitopes and have been established to neutralize pseudotype and live MERS-CoV [150,162]. None of the nAbs have undergone clinical trials except for (SAB-301) ii (8) MERS-CoV nAbs isolated from transchromosomic cattle [150].

### 7.5. Anticoagulant Therapies

Evidence from studies suggests that severe cases of SARS-CoV-2 disease may develop hypercoagulable conditions, especially thrombotic disease including acute coronary syndrome (ACS), venous thromboembolism (VTE) such as deep vein thrombosis (DVT) or microvascular thrombosis in the pulmonary vasculature/pulmonary embolism (PE) or stroke, elevated D-dimer levels, and high fibrinogen levels. Moreover, patients with underlying cardiovascular disease are at risk of morbidity and mortality. Anticoagulants and or antiplatelet medications, such as unfractionated heparin (UFH) and low molecular weight heparin (LMWH) were employed for the treatment of this group of individuals. However, management of these medications could be difficult in potentially critically ill patients [163,164].

### 7.6. Immunotherapy

Interleukin-6 (IL-6) is often referred to as a pleiotropic cytokine because of its known effects in regulating inflammatory reactions, immune responses, and infection [165]. However, overproduction of IL-6 could result in autoimmune diseases, such as rheumatoid arthritis (RA), juvenile idiopathic arthritis (JIA), and inflammation, which causes lung damage in COVID-19 patients [166]. In most cases of critically ill patients of COVID-19 disease, acute respiratory distress and multi-organ dysfunction are among the leading causes of death; therefore, an agent blocking IL-6 actions was proposed as a therapy for the treatment of COVID-19 disease [165,167]. Sarilumab, a typical IL-6, was found safe and effective in a specific subset of patients with hyperinflated COVID-19 pneumonia, but the study suggested that quantification of lung consolidation should be considered when treating patients sickened by COVID-19 with IL-6 blocking agent, also in clinical trial designs [168]. Another study also investigated the use of tocilizumab as an agent blocking IL-6 but found no significant difference in the death rate among patients treated with tocilizumab and patients who did not receive tocilizumab during treatment. Faster recovery was observed in a subset of patients showing minor lung inflammation at baseline. Moreover, no comprehensive secondary effects such as bleeding, thrombosis, or infections, and significantly less frequent invasive ventilation due to tocilizumab were observed [165,167,168,169]. However, there are opinions that the clinical use of IL-6 antagonists’ agent in autoimmune conditions is associated with an increased risk of severe and opportunistic infections. This observation raises the concern that IL-6 blockade, in addition to being unlikely to benefit COVID-19 patients, may also prove harmful. Therefore, more investigations are needed to validate the efficacy and suitability of IL-6 blockade for treating COVID-19 disease [170]. Also, corticosteroids including dexamethasone and glucocorticoids (prednisone, methylprednisolone, and hydrocortisone) are another type of anti-inflammatory drug that are usually used to treat rheumatologic diseases, like rheumatoid arthritis, lupus or vasculitis (inflammation of the blood vessels [171]. Daily dose equivalence of dexamethasone (6 mg,) prednisone (40 mg), methylprednisolone (32 mg), and hydrocortisone (160 mg) administered orally or intravenously reduced the risk of all-cause of mortality and duration mechanical ventilation [172,173]. Systemic corticosteroids compared with no corticosteroid therapy probably reduce the risk of 28-day mortality in critically ill patients with COVID-19 (moderate certainty evidence; relative risk 0.80 (95% confidence interval 0.70 to 0.91); absolute effect estimate 87 fewer deaths per 1000 patients (95% CI 124 fewer to 41 fewer)). In patients with severe COVID-19, systemic corticosteroids also probably reduce the risk of death (moderate certainty evidence; relative risk 0.80 (0.70 to 0.92); absolute effect estimate 67 fewer deaths per 1000 patients (100 fewer to 27 fewer) [123]. Recently, WHO recommended systemic corticosteroids for the treatment of patients with severe and critical cases of COVID-19 [123].

### 7.7. Vaccines

Effective control of the current SARS-CoV-2 pandemic is subject to the availability of appropriate vaccine(s) that could prevent viral shedding, transmission, and reduce disease severity. Several vaccines were tested in animals against MERS/SARS-CoV, including viral vectors, subunit vaccines, inactivated virus, protein vaccines, and recombinant DNA [35]. Presently, there are many auspicious target points for SARS-CoV-2, but further laboratory and clinical proof could be examined. The vaccine development strategies geared toward developing effective vaccines against the COVID-19 pandemic include DNA, mRNA in lipid nanoparticles, recombinant vectors, protein subunits and inactivated viruses. Several scientists across the globe are working with the WHO on about 174 potential vaccine candidates [112]. About 69 are in human clinical trials. About 20 are in the final stages of testing, while at least 89 preclinical vaccines are undergoing active investigation in animals (https://www.nytimes.com/interactive/2020/science/coronavirus-vaccine-tracker.html) accessed on 11 February 2021. As of 11 February 2021, about three vaccines including tozinameran, an mRNA vaccine (Pfizer-BioNTech); Moderna, an mRNA vaccine (Boston-based company Moderna); and AZD1222 (Covishield in India), and adenovirus were approved by certain national regulatory authorities for possible treatment for COVID-19 disease. The New York-based Pfizer and German company recorded over 95% effectiveness of their coronavirus vaccine, Moderna recorded 94.5% and up 90% by the University of Oxford and the Swedish company AstraZeneca vaccine. Other vaccines at various approval stages are presented in Table 4.

### 7.8. Current SARS-CoV-2 Treatment

There is no available clinically certified antiviral agent currently in use to treat infections conditioned by SARS-CoV-2 as well as SARS-CoV and MERS-CoV [110,147,174]. The management strategies of HCoVs are based on supportive care including broad-spectrum antibiotics, conservational fluid management, and supplemental oxygen therapy [16]. Previous studies on the molecular mechanisms of HCoVs [174,175] and the genomic structure of SARS-CoV-2 [176] revealed numerous potential targets where existing therapeutic antiviral agents could be redeployed for successful interventions.

### 7.9. Traditional Remedies

In recent times, there have been several claims of effective traditional herbal treatment for SARS-CoV-2. Traditional medicine of Chinese origin appears to show some efficacy in symptomatic care. Several prescriptions of Chinese traditional medicine were announced by the medical institutions and local government, including lung clearing and detoxification decoction [177]. Previous studies revealed that Shuanghuanglian oral liquid inhibited SARS-CoV-2, which could be mostly dependent on the activity of the bioactive components (baicalin, chlorogenic acid, and forsythin) having a certain inhibitory effect on viruses and bacteria [178,179]. The explanation of the therapeutic mechanisms of the components could be based on the effective reduction of the body inflammatory response caused by viruses and bacteria [180]. The broad-spectrum effect of lianhuaqinwen capsules on several influenza viruses, including H7N9, having the ability to reduce the level of inflammatory factors and regulate the virus in the early stage of infection was also observed [181]. In India, the Unani traditional system of medicine, which is an integral part of the national health system and was officially named “Unani medicine”, using natural herbal drugs, mineral, and animal sources for treatment, could find application in controlling the COVID-19 pandemic. It adopts several procedures such as isolation, quarantine, herbal purification or fumigation of surroundings, health-promoting and immune-modulating techniques, and the use of health-protective and symptom-specific drugs [182]. Drugs such as loban, sandroos, and za’fran vinegar are rich in several pharmacological bioactive ingredients based on scientific investigations [182].

Furthermore, Madagascar projected a coronavirus herbal cure (COVID organics) synthesized from the Artemisia plant [183]. All these traditional remedies still suffered approval setbacks from the WHO due to non-scientific evidence-based explanations supporting their efficacy. Herbal medications could also pose severe health threats due to hepatotoxicity, toxic contaminants (pesticides and heavy metals), and non-existing accurate prescriptions. As such, herbal formulas contain assemblages of several herbs, and the precise proprietary formulas are not available [181,183].

## 8. Control of Human Coronaviruses

All cases of infection and recent outbreaks revealed coronaviruses as a constant threat to human health and the economy as they suddenly emerge, spread easily, and lead to catastrophic outcomes. Understanding the source, pathogenesis, transmission dynamics, and identifying the intermediate host and factors contributing to the rapid spread of HCoVs are essential to the control of spread from animal to human and the continuous spread among humans. In the meantime, strict adherence to precautionary measures still holds despite the development of effective vaccines for the control of the endemic HCoVs, particularly SARS-CoV-2. Early case identification, diagnosis, and isolation; ideal care to patients infected; and the invention of efficient diagnostic, prophylactic and therapeutic strategies, including vaccines [184], are critical to mitigate the continuous spread of the ongoing pandemic. The WHO guidelines for COVID-19, adapted from MERS-CoV infection prevention and control, advocate proper regular personal hygiene practices, avoiding animal contact, drinking raw milk, and avoiding eating improperly prepared or contaminated foods [185]. Reports of hospital transmission suggest that all healthcare workers should strictly adhere to appropriate infection control practices including appropriate use of personal protective equipment (PPEs), droplets precautions during aerosol-generating procedures, and proper airborne infection isolation precautions when taking care of all category of patients [185]. To further limit community and international transmission, social distancing, restrictions on public gatherings, avoiding crowded areas, and limiting unnecessary local and international travels [3], could be the hard way to go until vaccine intervention is fully achieved.

## 9. Conclusions and Recommendation

Human coronaviruses have caused three significant outbreaks in the last two decades, including the current COVID-19 pandemic. The main symptoms at the onset of infection include cough, fever, and fatigue, among others, and bats were recognized as the presumptive natural reservoir. Viruses, especially SARS-CoV, MERS-CoV, and SARS-CoV-2, are extremely infectious, but the latter, which is accountable for the current worldwide pandemic, is more infectious and highly transmissible. While some cases are potentially fatal, SARS-CoV-2 poses severe public health implications and safety risks globally. Controlling the spread and reducing the morbidity and mortality of the pandemic is a major global challenge. As such, the ability of coronaviruses to evolve rapidly due to high rates of genomic substitution, recombination, and the ability for independent species crossing events, could suggest that vaccine intervention alone may not be effective in combating coronavirus outbreaks as there are chances for an entirely new different strain evolving as was the case with the SARS-CoV outbreak in late 2003–2004 by a different isolate [186]. Despite recent breakthroughs in vaccine development, the emergence of new variants of the SARS-CoV-2 suggests that a rapid approach and more attention are required for vaccine administration to combat the COVID-19 pandemic as soon as possible. More needs to be done to educate and sensitize people on the need to adopt the habit of general good personal hygiene practices, social distancing, and using existing drugs with potential antiviral effects against SARS-CoV-2 to manage disease progression, which has negatively impacted human social, economic, and political activities globally. There are possibilities of a future epidemic caused by SARS-like coronaviruses originating from different reservoir hosts at different times and locations based on the location and spread of the transmitting host. The identification of a diverse group of SARS-like coronaviruses in bats suggests chances of recurring future episodes. Therefore, intensive investigations to unravel and validate the possible reservoirs, the intermediate host, as well as the explanation about the ability of the virus to break the species barrier are needed to prevent future viral spillovers and possible outbreaks. Prompt mobilization and deployment of resources by concerned health organizations to source-track and identify new infectious agents and the possible reservoirs are crucial to mitigating the global impact of infection outbreaks.

## Figures and Tables

**Table 1:** Reported COVID-19 cases, recoveries, and deaths by continents.

Case Distribution	Continents					
	Europe	North America	Asia	South America	Africa	Oceania
**Cases**	48,859,923	40,985,581	57,556,562	33,955,392	5,971,777	80,818
**Recoveries**	45,859,625	34,444,421	54,645,672	31,155,314	5,185,839	69,782
**Deaths**	11,113,034	924,509	819,945	1,034,954	151,743	1318

**Table 2:** SARS-CoV-2 variants of concern.

Lineage	Variant Name	Characteristics
B.1.1.7	501Y.V1	First detected in Britain in December 2020 and about 50% more infectious.
B.1.351	501Y.V2	First identified in South Africa in December 2020 and shown the ability to reduce vaccine effectiveness leading to suspension of the AstraZeneca vaccine.
P.1	501Y.V3	First identified in Brazil in November 2020 with a similar mutation to B.351.
B.1.427, B1.429	CAL.20C	Found to be 20% more infectious, common in California. It carries the L452 mutation but does not appear to spread rapidly like B.1.1.7.
B.1.617	G/452R.V3	First detected in Maharashtra, India on 5 October 2020.

**Table 3:** Clinical manifestation, transmission routes and detection of human coronaviruses (HCoVs).

	SARS-CoV-2	MERS-CoV	SARS-CoV	HCoV-HKU1	HCoV-NL63	HCoV-229E	HCoV-OC43
Transmission	Fomites, faecal-oral, respiratory droplets.	Fomites, respiratory droplets.	Fomites, faecal-oral, respiratory droplets.	Fomites, respiratory droplets.	Fomites, respiratory Droplets.	Fomites, respiratory droplets.	Fomites, respiratory droplets.
Incubation period (days)	3–14	2–13	4–6	2–4	2–4	2–5	2–5
Clinical manifestation	High fever, Malaise, Dry cough, Sore throat, Fatigue, Sneezing, body pain, Myalgia, Headache, Diarrhoea, Difficult breathing	Cough, Sore throat, Shortness of breath, Malaise, Fever, Dyspnea, Chills, Myalgia, Arthralgia, Pneumonia, Diarrhea and vomiting, Rhinorrhea, Acute renal Impairment.	Fever, Headache, Malaise, Dyspnea, Myalgia, Dry cough, Respiratory, distress Diarrhoea, Hypoxemia, Pneumonia.	Fever, Dyspnea, Cough, Running nose.	Cough, Fever, Tachypnea, Rhinorrhea, Croup, Hypoxia.	Headache, Malaise, Sneezing, Nasal Discharge, Fever, Sore throat, Cough.	Headache, Malaise, Sneezing, Nasal, discharge, Fever, Sore throat, Cough.
Epidemiology	2019–2020 in China, Global spread thereafter	2012 in the Middle East 2015 in South Korea frequent in Middle East	2002–2003, in China, Global spread	Global, climax in winter	Global, climax in winter	Global, climax in winter	Globally climax in winter
% case fatality	3.4 [40]	34.40	11	-	-	-	-
Diagnostic methods	Point-of-care Testing of IgM/IgG, ELISA, STOP, CT scan, Real Time qPCR, deep sequencing [73,87,88,89]	pan-CoV RT-PCR, EIA, CT, scan, sequencing [20,87,88].	Reverse Transcriptase-PCR, EIA, sequencing [20,87,96]	RT-PCR, sequencing [54,90].	RT-PCR, sequencing [65,87].	RT-PCR, Sequencing [67,87].	RT-PCR, sequencing [9,97].

Key: POCT—Point-of-care Testing of IgM/IgG, ELISA—enzyme-linked immunosorbent assay, CT scan—computerized tomography, RT-qPCR—reverse transcriptase quantitative polymerase chain reaction, RT-PCR—reverse transcriptase polymerase chain reaction, STOP—SHERLOCK Testing in One Pot, EIA—enzyme-linked immunoassay or indirect immunofluorescence assay.

**Table 4:** List of leading coronavirus vaccines at the early approval for COVID-19.

Vaccine Name/Developer	Type	Phase (Clinical Trials)	Recent European and National (UK) Approval
Pfizer-BioNTech	mRNA	3	Emergency use in the U.S., E.U., and other countries. Approved in several countries.
Moderna	mRNA	3	Approved in Switzerland. Emergency use in the U.S., U.K., E.U., others.
Oxford-AstraZeneca	Adenovirus	3	Emergency use in the U.K., E.U., others. Approved in Brazil
Gamaleya	Adenovirus (Ad26, Ad5)	3	Emergency use in Russia, other countries.
CanSino	Adenovirus (Ad5)	3	Approved in China, emergency use in other countries.
Johnson &Johnson	Adenovirus (Ad26)	3	Emergency use in U.S., E.U., others.
Vector Institute	Protein	3	Approved in Turkmenistan. Early use in Russia.
Novavax	Protein	3	
Sinopharm	Inactivated	3	Approved in China, U.A.E., Bahrain. Emergency use in other countries.
Sinovac	Inactivated	3	Approved in China. Emergency use in other countries.
Sinopharm-Wuhan	Inactivated	3	Approved in China, limited use in U.A.E.
Bharat Biotech	Inactivated		Emergency use in India, other countries
